# Severe diphtheria with neurologic and myocardial involvement in a Swedish patient: a case report

**DOI:** 10.1186/s12879-018-3264-9

**Published:** 2018-07-31

**Authors:** Sten Skogmar, Johan Tham

**Affiliations:** 1 Department of Translational Medicine, Clinical Infection Medicine, Lund University, Skåne University Hospital, Malmö, Sweden; 2 Infectious Diseases Unit, Skånes University hospital, 205 02 Malmö, Sweden

**Keywords:** Diphtheria, Corynebacterium diphtheriae, Respiratory disease, Neurological manifestations, Myocarditis, Tuberculosis, Immunization

## Abstract

**Background:**

Diphtheria is caused by *Corynebacterium diphtheriae*. Although waning in incidence diphtheria can cause severe disease as in this rare Swedish case with several complications.

**Case presentation:**

A 55-year old male presented to the emergency room with severe respiratory symptoms and greyish membranes in the airways, which turned positive for *C. diphtheriae*. He was put on ventilator support and remained hospitalized for three months. During care he developed myocarditis and severe neurological disease and he was also co-infected with tuberculosis. The patient was discharged with a favorable outcome.

**Conclusions:**

Diphtheria should be suspected in patients with life-threatening pneumonia especially if the patient has a history of travelling. Our patient was not treated with diphtheria anti-toxin (DAT) which may have contributed to the severity of the disease.

## Background

Diphtheria is a rare disease in the western world, almost 100,000 diphtheria cases were reported in 1950 worldwide, while only around 8000 in 2009 and 4530 in 2015 according to World Health Organization (WHO) as a result of the improved immunization coverage [1]. In Europe during 2009, a total of 16 cases of diphtheria (mostly from Latvia) was reported to European Centre for Disease Prevention and Control (ECDC) but only three cases were reported in the United States between 2000 and 2007 [3]. Since a small outbreak in 1984, there have been only a few reported cases in Sweden. In the mid-1990s a larger outbreak was reported in former Soviet Republic states [4].

Patients with diphtheria usually develop cutaneous or respiratory disease but systemic manifestations such as; myocarditis, damage of the nervous system and kidney injury can be seen. This pathogenesis is related to an exo-toxin mediated from the *tox* gene which causes cell death [2]. Although the toxin does not have a specific target cell, damages in the myocardium and peripheral nerves are the most common complications. It has been estimated that 10–20% of patients with diphtheria have myocarditis and / or peripheral nerve neuropathy. The severity of the respiratory tract symptoms are related to the prevalence of these conditions. Up to two-thirds of the patients with severe diphtheria may present with myocardial and neurological complications and cardiac involvement is the major diphtheria related cause of death [5, 6].

## Case presentation

A fifty-five-year-old man with hypertension and who used regular medications for anxiety came to the Emergency Room in Malmö, Sweden, complaining of dyspnea in 2009. He had arrived to Sweden by plane from Sri Lanka five days prior, where he resides a large part of the year. Upon exiting Sri Lanka, he was put into custody for almost 6 weeks under unhygienic conditions.

**Fig. 1:**
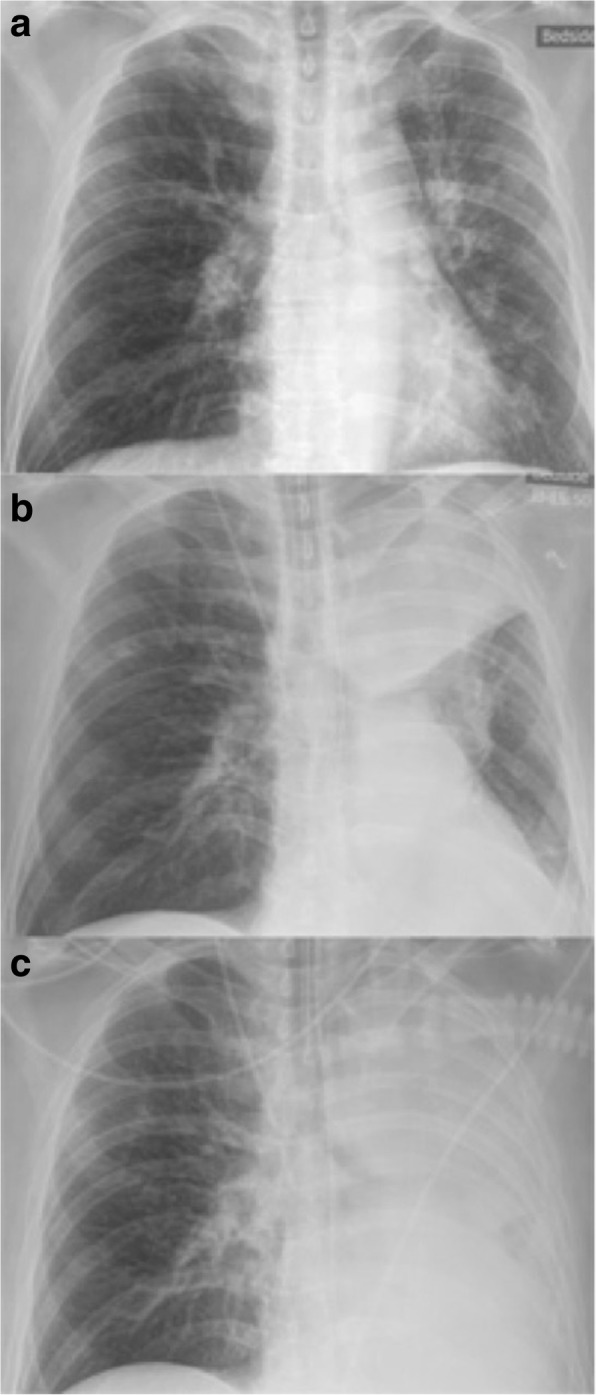
Serial chest radiographs during the first day showing a progressive atelectasis of the left lung. **a**. Taken at 10.30 in the morning (**b)**. 16.00 in the afternoon and (**c**). 23.50 at night

Already in Sri Lanka, but aggravated upon the return to Sweden, the patient experienced throat pain and shortness of breath. At the emergency room the patient presented with severe shortness of breath and fever of 39 °C. Throat inspection revealed swelling and greyish membranes. The patient deteriorated quickly with hypoxia and hypercapnia. He was intubated and put under ventilator support. Bronchoscopy showed greyish membranous plaques covering the larger part of the bronchus and partly occluding the left major bronchus. Serial X-rays showed progressive atelectasis of the left lung (Fig. 1). The membranes could mechanically be removed from its underlying layer and repeated bronchoscopies with lavage were performed.

Culture specimens were sent from larynx and bronchoscopy specimens as well as from a 1 cm^2^ skin ulceration. Due to suspicion of diphtheria, Loeffler’s tellurite media was used for culture. On day four from admission, the results from cultures showed growth of toxin producing *C. diphtheriae*, subsequently typed to *non-gravis*, both from the ulcer and from the respiratory tract. Serologies for Human Immunodeficiency Virus (HIV), hepatitis B and C and syphilis returned negative, as well as urine antigen tests for *Streptococcus pneumoniae* and *Legionella pneumophila*. The diphtheria strain was susceptible to both cefotaxime and erythromycin, which the patient was receiving since admission. It was in this situation judged too late for administration of DAT in relation to possible side effects and the duration of symptoms, and the patient was never administered this treatment. In the following weeks (day 5–14), the patient showed signs of improvement in infection control with decreasing C-reactive protein (CRP) and was afebrile, however the left lung remained deflated. The patient was under ventilator support for one month through tracheostomy performed on day seven. The prolonged time in ventilator was due to inability to recruit the left lung, despite repeated bronchoscopy and cleaning of greyish debris from the airways. A pleural catheter was also placed in the left inter pleural space with clearance of about one liter of transudative fluid. Bacterial cultures from the pleural fluid were negative.

**Fig. 2:**
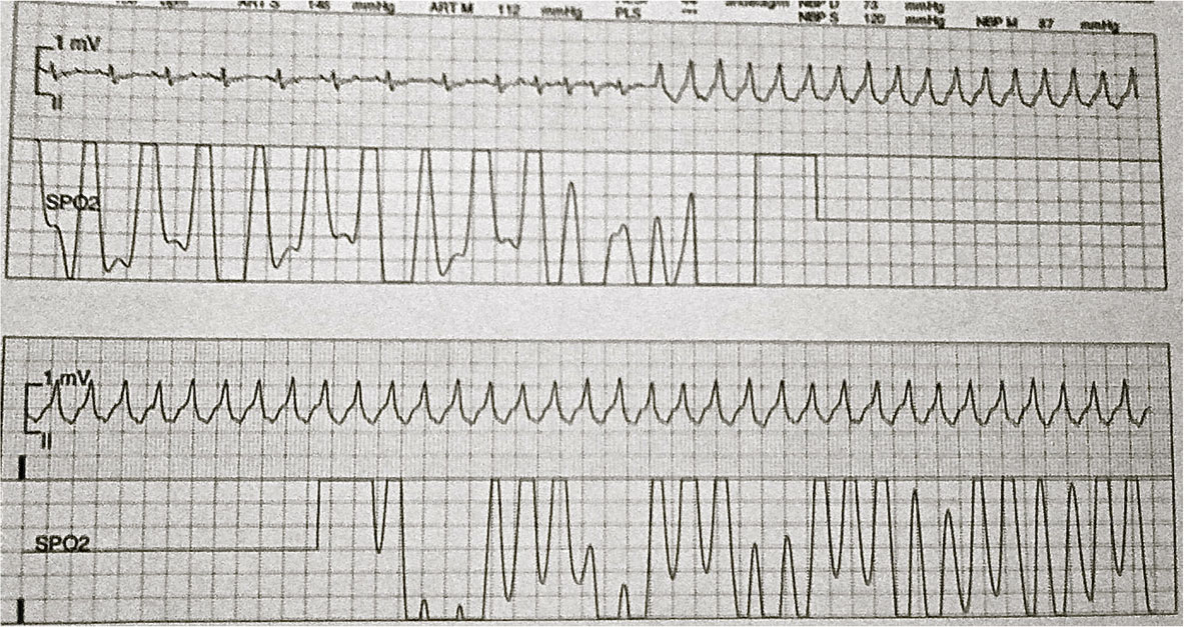
Patient developed Ventricular Tachycardia during the third week of admission

Between day 14 and 16 the patient had changes in the electrocardiogram with T-wave inversions and short periods of ventricular tachycardia as well as elevated cardiac enzymes that resolved spontaneously (Fig. 2). An echocardiogram was performed with no significant pathology. Additionally, a passing increase in serum creatinine was noted during this period. No clear reasons for these adverse organ effects were identified and were judged to be Diphtheria toxin related. On day 19 the patient deteriorated with fever and increased purulent secretions from the airways. This was considered due to Ventilator Associated Pneumonia (VAP) with Methicillin Resistant *Staphylococcus aureus* (MRSA) which was successfully treated with intravenous vancomycin for two weeks. In the fourth week, the patient improved and could gradually be weaned off of the ventilator. The tracheostomy tube was removed on day 46 from admission and the patient was mobilized in the following week with physical therapy and was prepared to be discharged to a center for rehabilitation.

On day 55, however, the patient developed a gradual onset of neurological symptoms. First he got increasingly weaker voice and shortly thereafter weakness of the extremities, and increasing difficulties to breathe. From day 58, the patient quickly deteriorated with a complete paraplegia and respiratory failure ensued, requiring reintubation. Repeated neurographic examinations showed severe polyneuropathy with mixed demyelination and axonal loss. The pattern was not deemed to be consistent with Guillain-Barré, nor Critical Illness and was judged to be due to toxin effects of diphtheria toxin thus no neuroimaging was considered necessary. Furthermore, lumbar puncture showed no significant pathology.

On day 56, surveillance Bronchiole Alveolar Lavage (BAL)-culture was sent for extensive testing and culture due to respiratory deterioration. This subsequently showed growth of *Mycobacterium tuberculosis* (MTB) fully sensitive to rifampicin, isoniazide, pyrazinamide and ethambutol. MTB-PCR was negative. Bronchoscopy specimens for MTB culture and PCR had previously been sent for investigation on day two from admission, but was negative at that time. The patient was started on combination therapy for pulmonary tuberculosis (TB) on day 70.

**Fig. 3:**
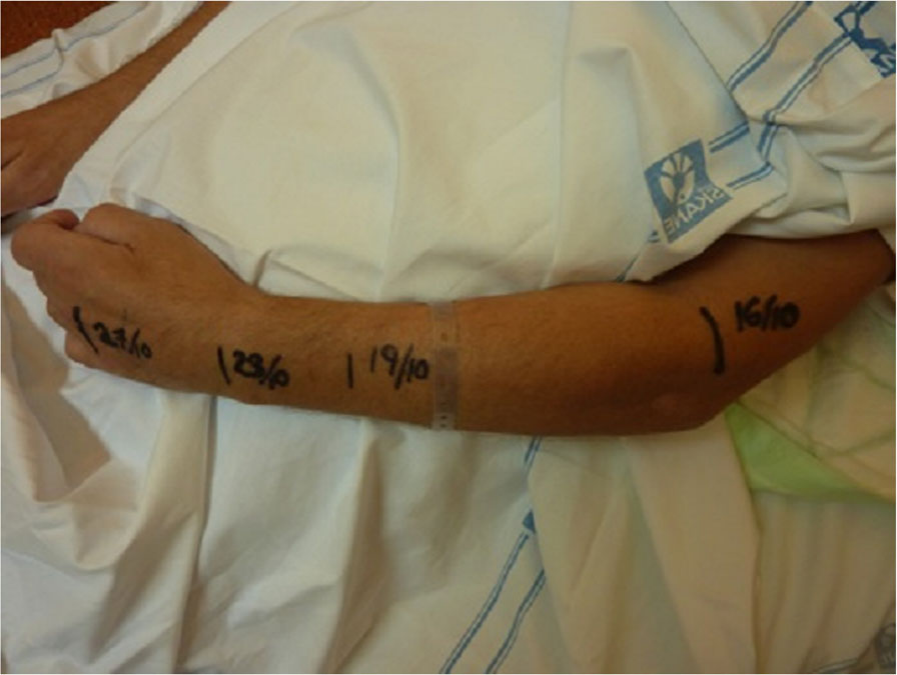
Gradual return of sensory function over the course of 11 days. The lines were drawn on the patient to mark the recovery of skin sensation

The patient gradually regained motor function from day 80 and the patient could once again be weaned off of the ventilator. The sensory functions likewise gradually returned from the center to the periphery (Fig. 3). After an extended stay for mobilization, the patient was discharged to a rehabilitation clinic on day 91 from admission. On follow up after three and six months, the patient continued to improve in motor and sensory functions, The patient was followed for three years reporting only minor polyneuropathic symptoms in his feet, but had resumed his daily activities with no motor impairments.

## Discussion

Our patient displayed the full range of clinical findings associated with diphtherial disease [7]. At presentation, the main problem was respiratory failure due to left sided lung collapse likely due to diphtherial membranous occlusion of the left main bronchus. It was early suspected that diphtheria might be the cause which provided a diagnosis within the first four days. This aided in the further management of the patient; in our microbiological department, no routine screening for *C. diphtheriae* is performed as it requires culture on media containing tellurite and communication of the clinical suspicion to the microbiological clinic proved crucial to establish the early diagnosis, which was also pressed on in another Swedish case of diphtheria [8].

In the first month, the patient had signs of both myocardial and kidney impairment which have been well documented effects ascribed to exo-toxin [9]. Furthermore our patient also developed fulminant neurological symptoms. Since our patient was in ventilator support we could not evaluate any neurological symptoms from the pharynx, larynx or palate. These involvements have been described to develop earlier after initial diagnosis (1–5 weeks) of diphtheria [10]. We could however observe the development of peripheral nerve symptoms which occurred from day 55 when the patient had been extubated. Yet late, this is within the timeline for the development of peripheral nerve demyelization due to diphtheria described in several articles [10–12] [7, 10, 11].

Current CDC guidelines recommend administration of DAT to all laboratory confirmed cases with respiratory diphtheria after testing for hypersensitivity to horse serum since about 10% get serum sickness but rarely anaphylaxis [13–15]. Our patient did not receive DAT, due to the critical state of the patient in the early stages of care. Also, the patient presented with several days of symptoms and the diagnosis was not established until day three from presentation and it is unclear if anti-toxin would have prevented the late onset symptoms. According to old studies DAT should be administered within a week to reduce mortality but the best results was seen when treated within 24–48 h [14].

Regarding vaccination status of the patient, it was never fully elucidated if he had been vaccinated or not, as neither he nor his family members remembered if it had been given to him. He did not believe he had been given boosters regularly. In Sweden, diphtheria toxin was introduced in the common immunization program after World War II, with some regional delays. Our patient was born in 1951 and may or may not have been given vaccination. Even if it had been given, studies have shown a waning immunity with age with protective titers of less than 50% in western materials [8, 16].

Additionally this patient had combined infection of diphtheria and pulmonary tuberculosis which has rarely been reported [17]. Negative culture and PCR from early bronchoscopy suggests that our patient activated a latent tuberculosis infection during care than presented with both initially.

## Conclusion

This patient case of infection with toxin producing *C. diphtheriae* non-gravis had all the described toxin complications of diphtherial infection. The patient made a near full recovery. This case illustrates the importance of vigilance already in the emergency setting for patients presenting with membranes in the airways to properly diagnose patients. It also shows that diphtheria may present with late onset of toxin effects, in this case 55 days upon admission and that prompt administration of DAT should be strongly considered when diphtheria is suspected.

## Abbreviations

BAL: Bronchiole Alveolar Lavage
CDC: Center for Disease Control
CRP: C-reactive protein
DAT: Diphtheria Anti-toxin
ECDC: European Centre for Disease Prevention and Control
HIV: Human Immunodeficiency Virus
MRSA: Methicillin Resistant *Staphylococcus aureus*
MTB: Mycobacterium tuberculosis
PCR: Polymerase chain reaction
TB: Tuberculosis
VAP: Ventilator Associated Pneumonia
WHO: World Health Organization
